# Role of B7 family members in glioma: Promising new targets for tumor immunotherapy

**DOI:** 10.3389/fonc.2022.1091383

**Published:** 2023-01-18

**Authors:** Yan Wang, Mengxi Li, Gang Wang, Hui Wu

**Affiliations:** ^1^ Department of Radiation Oncology, Third People’s Hospital of Zhengzhou, Zhengzhou, Henan, China; ^2^ Department of Radiation Oncology, Affiliated Cancer Hospital of Zhengzhou University, Zhengzhou, Henan, China

**Keywords:** glioma, PD-L1, PD-L2, B7-H3, B7-H4, B7-H6

## Abstract

Glioma, is a representative type of intracranial tumor among adults, usually has a weak prognosis and limited treatment options. Traditional therapies, including surgery, chemotherapy, and radiotherapy, have had little impact on patient survival time. Immunotherapies designed to target the programmed cell death protein 1 (PD-1)/programmed death ligand 1 (PD-L1) signaling pathway have successfully treated various human cancers, informing the development of similar therapies for glioma. However, anti-PD-L1 response rates remain limited in glioma patients. Thus, exploring novel checkpoints targeting additional immunomodulatory pathways for activating durable antitumor immune responses and improving glioma outcomes is needed. Researchers have identified other B7 family checkpoint molecules, including PD-L2, B7-H2, B7-H3, B7-H4, and B7-H6. The current review article evaluates the expression of all 10 reported members of the B7 family in human glioma using The Cancer Genome Atlas (TCGA) and the Genotype-Tissue Expression (GTEx) data, as well as summarizes studies evaluating the clinical meanings and functions of B7 family molecules in gliomas. B7 family checkpoints may contribute to different immunotherapeutic management options for glioma patients.

## 1 Introduction

Glioma is a representative tumor regarding central nervous system (CNS), and accounts for approximately 81% of adult primary brain tumors (1). Based on World Health Organization (WHO) Classification updated in 2016, glioma treatment and prognosis can vary dramatically (2). Conventional treatment modalities for glioma patients include surgery, radiotherapy, and chemotherapy. While these options have achieved remarkable progress in recent decades, glioma patient survival rates remain low, especially among those with glioblastoma (GBM). Thus, new treatment strategies or agents shall be developed urgently.

Immunotherapy is a revolutionary cancer treatment that targets checkpoints in various solid tumors, including gliomas (3). T cells are pivotal effectors in the immune response to cancer, and the loss of function of this cell type can promote immune evasion (4). Immune responses are under the strict controlling of the B7 family memembers, including co-stimulatory molecules and co-inhibitory molecules. Co-stimulation can be balanced by co-inhibitory signals, that determine the activation or the inhibition of T cells (5). B7 family members also can essentially regulate the tumor progression, growth, proliferation, invasion, and drug sensitivity (6). Thus, the B7 family has received particular attention for their potential role as immune checkpoint inhibitors (ICIs) in cancer treatment. By now, there have been ten identified B7 family molecules: B7-1 (CD80), B7-2 (CD86), B7-H1 (CD274, PD-L1), B7-DC (CD273, PD-L2), B7-H2 (CD275), B7-H3 (CD276), B7-H4 (B7x, B7S1or VTCN1), B7-H5 (GI24, VISTA or PD-1H), B7-H6 (NCR3LG1) and B7-H7 (HHLA2) (7).

**Figure 1:**
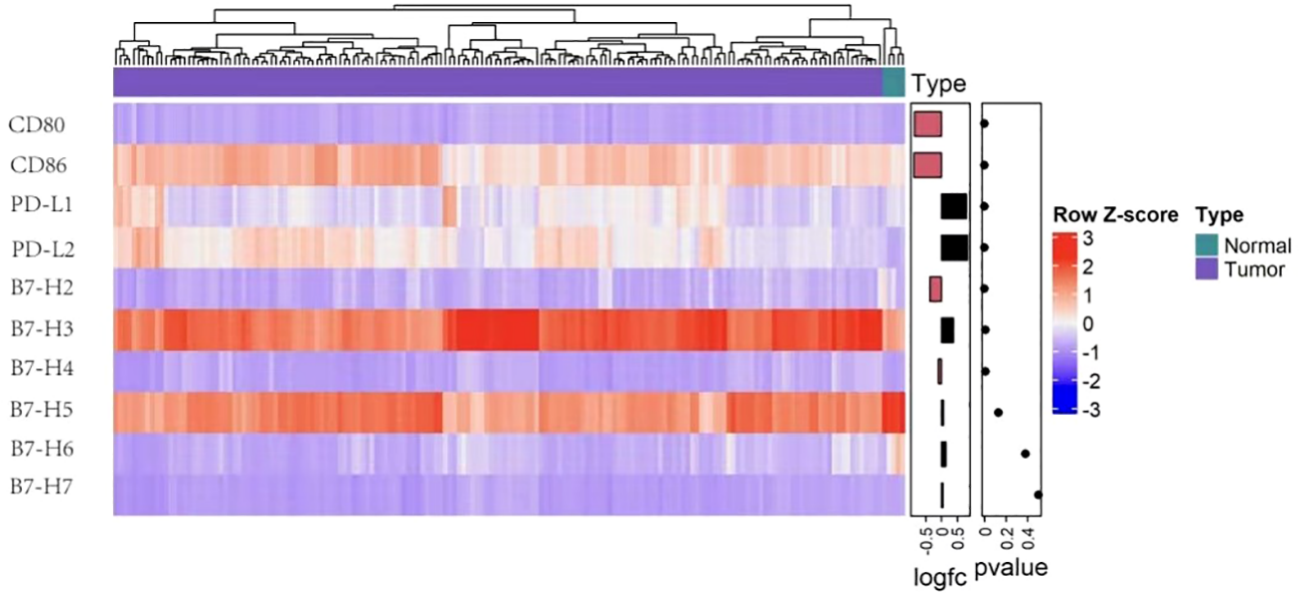
B7 family molecules expression levels in glioma. Heatmap representing expression of the ten B7 family member genes in normal (n = 5) and glioma tissue (n = 173) in TCGA (n=173). The data came from UCSC Xena and *t* test served for the analysis after the log2 transformation.

Several B7 family members are highly expressed in glioma, suggesting that these molecules participate the anti-glioma immune response (8, 9). Multiple mechanisms regulate the expression of B7 molecules. Blocking B7 activates T lymphocytes and NK cells and restores antitumor immunity (10). The current study used TCGA and GTEx data to investigate the expression of different members of B7 family in glioma. B7-H3 and B7-H5 presented a higher expression than other family members, suggesting these two molecules may play an essential role in anti-glioma immunity (**Figure 1**). This could explain the limited efficacy exerted by PD-1/PD-L1 therapy against this disease. Nevertheless, few studies have investigated the relationships between B7-H5, B7-H7 and glioma, respectively. The current review summarizes research on other B7 family members, including PD-L1(B7-H1), PD-L2(B7-DC), B7-H3, B7-H4, and B7-H6, in glioma. Further study shall be conducted on these molecules to develop new and useful immunotherapies, either single or combined medicines.

## 2 Clinical meanings and functions of PD-L1 in gliomas

Programmed death ligand 1 (PD-L1), also known as CD274 or B7-H1, was first named B7-H1 by Dong et al. in 1999 (11). PD-L1 can be encoded by the PDCDL1 gene, has seven exons, includes both IgV-like and IgC-like extracellular domains (12), and is the first functionally characterized ligand of coinhibitory PD-1. Multiple cancer types see the expression of PD-L1, including lung cancer, glioma, Merkel cell carcinoma, head and neck cancer (HNC) and classical Hodgkin’s lymphoma (CHL) (13–17). In gliomas, PD‐L1 expression ranges from 6.1–88% (18) and is mainly controlled by TLR, EGFR, and IFN signaling. TLR signaling promotes PD-L1 expression in gliomas by activating the MyD88/TRAF6/MEK/ERK pathway (19). EGFR is activated by tumor growth factor-α or EGF binding, inducing Ras/RAF/MAPK and PI3K/Akt-1/mTOR signaling and promoting the PD-L1 expression (8). PTEN, which negatively regulates the Akt activation, can vitally regulate the PD-L1 expression in glioma. Indeed, PTEN homozygous deletions or mutations are found in 36% of gliomas and correlate positively with PD-L1 expression (8). MicroRNA-34a (micR-34a) is also relate to the PD-L1 expression in gliomas, which modulates EGFR or PD-L1 translation for suppressing tumors (20). IFN type 1 (α, β, and ω) regulates PD-L1 by binding to the type 1 interferon receptor, and includes two subunits of IFNAR 1 and IFNAR 2 (21). Receptor binding induces the STAT 1–3 signaling cascade and the JAK1 and JAK2 activation, resulting in elevated PD-L1 expression (22). Meanwhile, *IFNAR1/2* gene silencing reduces PD-L1 expression.

**Figure 2:**
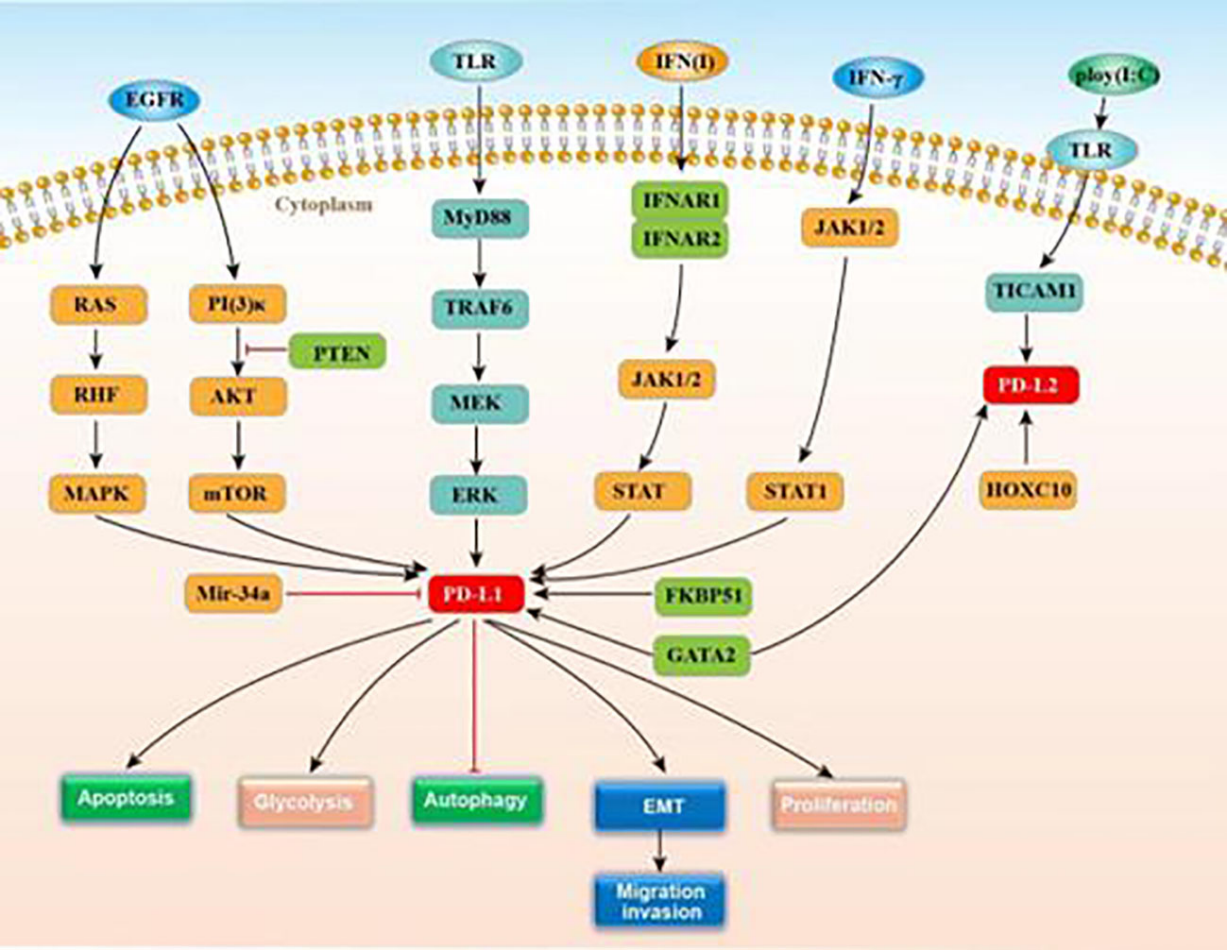
The function and regulatory mechanisms of PD-L1/PD-L2 in gliomas.

The molecular chaperone, FK506-binding protein 51(FKBP51), is an important biomarker of metabolic dysfunction and is abundantly expressed in glioma. D’Arrigo et al. reported that FKBP51s led to PD-L1 expression up-regulation on the plasma membrane through the catalysis of the protein folding needed for the later glycosylation, confirming it as an underlying target for GBM immunotherapy (21). According to Chen. et al., PD-L1/Ras/ERk signaling promotes the EMT, the migration, and the invasion of glioma cells (23). More accurately understanding the PD-L1 mechanisms of action could inform the development of new immunotherapies for glioma. **Figure 2** displays the known roles and regulatory mechanisms of PD-L1.

The PD-1/PD-L1 axis acts as a crucial checkpoint in cancer immune evasion and progression. The binding of and PD-1 and PD-L1causes T cell exhaustion, anergy and apoptosis, as well as reduces cytotoxicity (24). Using anti-PD-1/PD-L1 antibodies for treatment has a similar effect. In contrast, blocking the process of PD-L1 binding to PD-1 creates an immunosuppressive microenvironment and leads to T cell activation, to enable T cells recognize as well as kill tumor cells (25). Anti-PD-1 antibodies are applied to different solid tumors (26). Clinical trials of PD-1/PD-L1 inhibitors are ongoing in patients with glioma. CheckMate143 (NCT02017717), a randomized controlled clinical trial comparing Nivolumab (PD-1 antibody) with Bevacizumab in recurrent glioblastoma (rGBM) patients, is the first trial launched in the United States (27). At the 2017 WFNOS meeting, researchers reported that Nivolumab failed to prolong patients’ overall survival time (OS) compared with Bevacizumab. The Nivolumab group possessed obviously lower progression free survival time (PFS) relative to the Bevacizumab group (1.5 vs. 3.5 months, respectively) (27). However, in a phase II trial, single Nivolumab therapy induced an effective overall response rate (ORR) of 7.8% (27). In 2021, a multicohort phase 1b KEYNOTE-028 study (NCT02054806) comparing Pembrolizumab to PD-L1 positive GBM found that Pembrolizumab monotherapy promoted durable antitumor activity with a median PFS of 2.8 months and a median OS of 13.1 months. An ORR of 8% was observed (28). Most recently, a Phase III trial (NCT02667587) of Stupp regimen+Nivolumab or Placebo for newly diagnosed enzyme O(6)-methylguanine-DNA methyltransferase (MGMT) methylated GBM was reported. Regretfully, Nivolumab added to Stupp care did not improve survival in patients. The PFS was 10.6 months with Nivolumab + radiotherapy (RT) + temozolomide (TMZ) vs 10.3 months with Placebo+RT+TMZ and mOS was 28.9 months vs 32.1 months, respectively (29). These findings indicated that ICIs showed anti-tumor activity in in minor patients. This may be explained by several reasons. First, the tumor microenvironment of GBM contains few T-cells and instead is dominated by tumor-associated macrophages (TAMs), especially IDH-mut glioma (30, 31). Second, GBM is rich in myeloid-derived suppressor cells (MDSCs) (32), which can strongly suppress the activity of T cells, NK cells, and certain myeloid cells. Third, high tumor mutational load has rarely been observed in GBM (33). In addition, a large amount of TGF is present in tumor microenvironment, including TGF-β, L10, IDO and other immunosuppressive factors. At present, regulatory T (T-reg) cells are considered as one of the main reasons for the immunosuppressive microenvironment of GBM (34). Together, these findings indicate the immunologically “cold” nature of GBM.

Recently, studies suggested that radiotherapy could remodel tumor inflammatory environment and turn immunologically ‘cold’ to ‘hot’ (35). In a 2012 study by Zeng et al., they found that mice (models of GBM) that received Stereotactic Radiosurgery + anti-PD-1 therapy had a near doubling of mOS than that received anti-PD-1 therapy alone (36). Then, combining SRS with ICIs may provide an attractive combination for treating GBM. In 2019, a study by Cloughesy et al. found that compared with adjuvant pembrolizumab, neoadjuvant pembrolizumab plus adjuvant pembrolizumab confer a significant improvement in OS (13.7vs 7.5 months) and PFS (3.3vs 2.5months) for patients with rGBM (37). Thus, it may be a novel management paradigm for rGBM. A phase 2 clinical trial (NCT03197506) of neoadjuvant ICI therapy are ongoing in the Mayo Clinic. **Tables 1**, **2** list the completed clinical trial results and ongoing trial results, respectively.

**Table 1:** Ongoing clinical trials targeting B7 family molecules.

Target	Drug	Disease	Phase	N	Trial ID	Status
PD-L1	PD-L1 CAR-T	Glioma/Recurrence Tumor	1	100	NCT03423992	Recruiting
PD-L1	Avelumab	Glioma	1	60	NCT03893903	Recruiting
PD-L1	Atezolizumab	Glioma	1	18	NCT04160494	Recruiting
PD-L1	Atezolizumab	GBM	1	12	NCT05423210	Not yet recruiting
PD-L1	Olaparib /Durvalumab	Glioma /Cholangiocarcinoma /Solid Tumor	2	78	NCT03991832	Recruiting
B7-H3	B7-H3/CAR-T	Central Nervous System Tumor	1	90	NCT04185038	Recruiting
B7-H3	131I-Omburtamab	DIPG	1		NCT05063357	Not yet recruiting
B7-H3	B7-H3CAR-T	Brain and Nervous System	1	39	NCT05474378	recruiting
B7-H3	B7-H3CAR-T	GBM	1	36	NCT05366179	Not yet recruiting
B7-H3	B7-H3CAR-T	rGBM	1	12	NCT04385173	Unknown status
B7-H3	B7-H3CAR-T	GBM	1	30	NCT05241392	Recruiting
B7-H3	B7-H3 CAR-T	rGBM	1/2	40	NCT04077866	Recruiting

GBM, glioblastoma; recurrent glioblastoma, rGBM; N, number; DIPG, diffuse intrinsic pontine glioma; CAR-T, chimeric antigen receptor-T cell therapy.

**Table 2:** Completed clinical trials that target B7 family molecules in glioma.

Target	Drug	Disease	Phase	N	Trial ID	ORR/mPFS/mOS
PD-1	Nivolumab	r/High Grade Glioma/Brain Cancer	2	43	NCT03925246	NR
PD-1	DNX-2401/Pembrolizumab	Brain cancer	2	49	NCT02798406	NR
PD-1	Pembrolizumab	HGG		13	Lombardi et al. (38)	m PFS: 2.2 months m OS: 5.6 months
PD-1	Nivolumab	Glioma/GBM/Astrocytoma	1	6	NCT02529072	cohort1: Preoperative nivolumab and postoperative nivolumab + DC vaccine: (mPFS: 4.3 months mOS: 8.0 months) cohort2: Preoperative nivolumab + DC vaccine and postoperative nivolumab + DC vaccine (mPFS: 6.3 months mOS: 15.3 months)
PD-1	Nivolumab/Bevacizumab	GBM	3	369	NCT02017717	Arm A: Nivolumab, ORR: 7.8% Arm B: Bevacizumab, ORR: 23.1%
PD-1	nivolumab	GBM	II	29	NCT02550249	Presurgery nivolumab +surgery+ adjuvant nivolumab: m PFS: 4.1 months m OS: 7.3 months
PD-L1	Avelumab	GBM	1	13	NCT03341806	Completed, NR
PD-L1	Axitinib/Avelumab	rGBM/Glioma (WHO IV)	2	52	NCT03291314	Cohort1: (Low baseline corticosteroids) Axitinib + avelumab: ORR:33.3% mPFS: 12.0 weeks mOS: 10.7 weeks Cohort2: (High baseline corticosteroids): Axitinib+ avelumab after 6 weeks): ORR: 22.2%
PD-L1	Avelumab	GBM	2	6	NCT02968940	NR
PD-L1	Durvalumab/Tremelimumab	Glioma/rGBM	2	36	NCT02794883	NR
PD-L1	DSP-7888	GBM/DIPG	1/2	18	NCT02750891	NR
PD-L1	Atezolizumab	GBM	1	16	NCT01375843	ORR: 6% mPFS: 1.2 months mOS: 4.2 months
PD-L1	Durvalumab/Bevacizumab	GBM	2	159	NCT02336165	A: Newly diagnosed uMGMT: (Durvalumab + radiotherapy) mOS: 15.1 months B: Bevacizumab‐ naïve rGBM (B1: Durvalumab: 12‐months-OS: 44.4%; B2: Durvalumab + Bevacizumab: NR B3: Durvalumab + Bevacizumab: NR C: Bevacizumab‐recurrent: Durvalumab + Bevacizumab: mOS: 5.6 months

GBM, glioblastoma; recurrent glioblastoma, rGBM; high grade glioma, HGG; N, number; ORR, Objective response rate; mPFS, median progression free survival; mOS, median overall survival; NR, not reported; uMGMT, MGMT unmethylated.

Anti-PD-L1 antibodies, including Avelumab, Durvalumab, and Atezolizumab, enjoy wide application in clinical practice. Avelumab functions as a fully human IgG1 mAb which exerts selective blocking effect on PD-L1, as well as facilitates anti-tumor T-cell activity (26). A non-randomized, open-label phase II trial of Avelumab for rGBM treatment was completed in Belgium and elicited an ORR of 33.3% (39). According to a phase Ia trial, the PR and steady disease (SD) of Atezolizumab were 6% and 18%, respectively, in rGBM patients (40). In a separate phase II study (41), Durvalumab combined with standard or reduced dose Bevacizumab had no significant effect on a cohort of Bevacizumab-naïve rGBM patients (41). These findings suggest that current PD-L1 inhibitor treatments for patients with recurrent glioma are poor. **Tables 1**, **2** give the completed and the ongoing clinical trial results, respectively.

## 3 Clinical meanings and functions of PD-L2 in gliomas

PD-L2, also called CD273, is a receptor for PD-1. Like PD-L1, PD-L2 contains IgV-like and IgC-like extracellular domains and exists on multiple immune, endothelial, and tumor cells (42). Less is known about how PD-L2 is regulated than PD-L1. Fu et al. showed that *GATA-binding factor 2 (*GATA2) was capable of promoting the expressions of PD-L1 and PD-L2 (43). *GATA2*, encoding a zinc finger transcription factor required for normal hematopoiesis, is located on chromosome 3q21.2 (44). This transcription factor can increase the expressions of PD-L1 and PD-L2, which is needed for PD-L2 expression. Li et al. found that HOXC10, a which belonged to the *homeobox* genes (HOX) gene family, could considerably affect the physiological processes of mammalia. This gene is upregulated in glioma and promotes the expression of PD-L2, and other genes related to tumor immunosuppression (45). HOXC10 binds directly to PD-L2 promoter regions. De Waele et al. reported that poly (I:C) (Toll-like receptor 3 agonist, TLR-3) stimulates the expressions of PD-L1 and PD-L2 through TLR3-TICAM1 signaling (46). **Figure 2** displays the regulatory action of PD-L2 expression. Like PD-L1, PD-L2 crucially modulates T cell activation, proliferation, and immune escape by human tumors (47). In glioma patients, PD-L2 expression could report worse clinical outcomes (43). Thus, targeting PD-L2 signaling may serve as a potential substitute therapy for glioma.

## 4 Clinical meanings and functions of B7-H3 in gliomas

B7 homolog 3 (B7-H3), also named CD276, refers to a 316 amino acid long type I transmembrane protein (48). In 2001, researchers first clone it from a cDNA library from the dendritic cells (DCs) (49). The human B7-H3 gene can be observed on chromosome 15 (48). While B7-H3 mRNA presents an ubiquitous expression in various tissues and cells, B7-H3 protein can only be found in resting fibroblasts, osteoblasts, activated T lymphocytes, endothelial cells, NK cells, and APC (10). The expression of B7-H3 were assessed by immunohistochemistry and western-blot in human GBM and benign brain tissue, including 2IgB7-H3 and 4IgB7-H3 two isoforms (50, 51). Despite the presence of 2IgB7-H3 in benign brain tissue, 4IgB7-H3 showed certain expression in GBM. 2IgB7-H3 had a higher expression in rGBM tissue, more resistant to apoptosis under the mediation of temozolomide (9). A separate study found that 2IgB7-H3 mRNA presented expression in glioma tissues but was weak or undetectable in benign brain tissues. Meanwhile, 4IgB7-H3 mRNA could be found in benign brain and in glioma tissues (**Table 3**) (52).

**Table 3:** Two forms of B7-H3 expression in normal brain and glioma tissue.

	Forms	Normal tissue	Glioma tissue
mRNA	2IgB7-H3	–	+
	4IgB7-H3	+	+
Protein	2IgB7-H3	+	+
	4IgB7-H3	–	+

-, weak or undetectable; +, positive.

**Figure 3:**
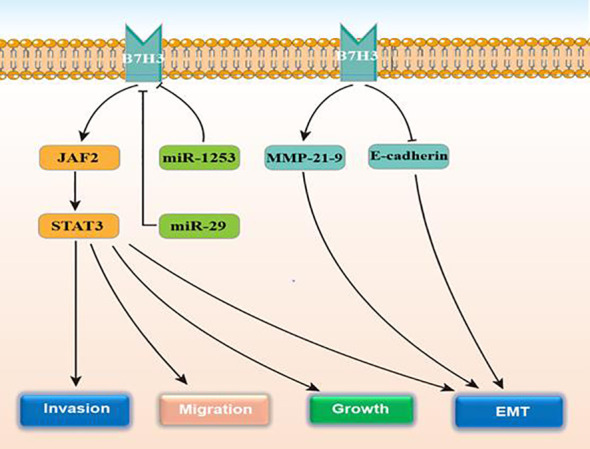
The function and regulatory mechanisms of B7-H3 in gliomas.

Glioma patients with isocitrate dehydrogenase (IDH) wild‐type or a higher tumor grade express more B7-H3 (53). Studies also show that microRNA‐29 family members can negatively regulate B7‐H3 in glioma tissue. B7-H3 is positively correlated with TLR signaling (53). This protein is present in many kinds of cancers, including glioma, and is relevant to tumor aggressiveness and reports poor prognosis (54, 55). According to Zhong et al., elevated B7-H3 expression exerted an obviously positive impact on the proliferation and invasion of glioma cells both *in vitro* and *in vivo*, that leads to weak clinical prognosis (56). Elevated B7-H3 levels results in the activation of the JAK2/STAT3 prosurvival signaling pathway, that contributes to tumor growth, meanwhile inducing EMT in cancer cells. In addition, B7-H3 induces tumor cell EMT processes by downregulating e-cadherin and upregulating MMP-2/-9 expression. The STAT3 inhibitor, NAP, can remarkably suppress the glioma growth and invasion and could thus be a potential strategy for treating glioma. MMP-2 (main) degrades the extracellular matrix and induces cell migration from the primary tumor to the surrounding environment. Exosomes are membrane vesicles that were released by cancer cells that promote cancer cell growth and increase tumor swelling, invasion, and migration (57) Recently, Ciprut et al. showed that angio-associated migratory cell protein (AAPP) was a binding partner of B7-H3 and that B7-H3-induced immunosuppression could be blocked by targeting AAPP (58). Kanchan et al. found that CD276 is an oncogenic target of miR-1253. MiR‐1253 transfection downregulates CD276 expression. However, tumor cell migration and invasion are substantially reduced when CD276 is silent (59). **Figure 3** displays the regulatory actions of B7-H3 expression.

Functionally, B7-H3 promotes tumor-immune escape and confers a more aggressive phenotype to multiple tumor cell types (60). The B7-H3 checkpoint can promisingly serve for cancer immunotherapy as a novel target. According to studies, using a monoclonal antibody to target B7-H3 can safely and effectively serve for treating stage IV childhood neuroblastoma (61). MGA271, an anti-tumor-associated B7-H3 monoclonal antibody, inhibits the growth of glioma cells through ADCC, thereby increasing the anti-tumor response (62). Meanwhile, 8H9 acts as a murine IgG1 mAb targeting B7-H3 (63, 64), which, based on the immunostaining, presents a broad response in human solid tumors, such as embryonal tumors and carcinomas (63). This mAb exhibits a good tumor uptake in xenograft models of both sarcoma and brain tumors (65).

Chimeric antigen receptor (CAR) T cells have become an useful immunotherapeutic approach in cancer treatment (66). CAR essentially constitutes CAR-T, relying on which T cells can recognize tumor antigens without needing HLA, and recognize a larger number of wide target antigens compared with natural TCR (67). As CAR-T cells has enjoyed a successful application to treating hematological malignancies, using CAR-T cell therapy for solid tumor is gaining more and more attentions (68). Many clinical trials are conducted in several countries including the US, China and Europe, and with the trail progress and outcome being strictly detected. To date, some preclinical and clinical studies regarding the CAR-T immunotherapy specific to gliomas have achieved good results (69–71). Tang et al. constructed B7-H3-specific CAR-T cells and evaluated it antitumor activities in primary glioma cells and GBM cell lines, as well as found that the CAR-T group of orthotropic GBM model has significantly longer survival time than that of control group (72). According to the study by Nehama et al. in 2019, B7-H3-specific CAR-T cells release effector cytokines like IL-2 and IFN-γ, meanwhile controlling the growth of neurospheres and human GBM cell lines (73). In consistent with Tang et al’s report, compared with control T cells, B7-H3 CAR-T group significantly prolonged the survival of treated mice. B7-H3-specific CAR-T has promising antitumor activities in immune-competent animal models and patient-derived orthotopic xenograft. Dual CAR-T target antigens improve variation of antigens and the heterogeneity in treating solid tumors and showed enhanced antitumor effects (74). Accordingly, B7-H3 is likely to be a promising CAR-T target for GBM. **Table 2** lists the ongoing clinical trial results. These findings confirm B7-H3 CAR-T as an useful and safe immunotherapeutic agent for tumors.

## 5 Clinical meanings and functions of B7-H4 in gliomas

**Figure 4:**
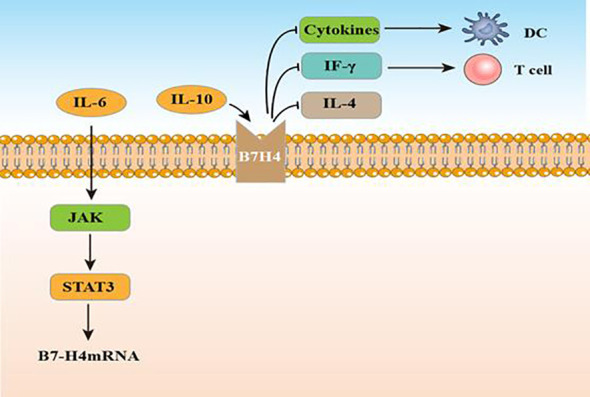
The function and regulatory mechanisms of B7-H4 in gliomas.

In 2003, B7 homolog 4 (B7-H4), also called B7x and B7S1, was identified by three laboratories as it was similar to other B7 family molecules (75). As a type I transmembrane protein, it can share 20–30% amino acid homology with other family members in its extracellular region. Similarity in B7-H4 amino acid sequences between mouse and human is approximately 87% (76). B7-H4 encodes the VTCN1 protein, which includes 283 and 282 amino acids in murine and humans, respectively (77). From the perspective of structure, B7-H4 possesses an extracellular, a hydrophobic transmembrane, together with an intracellular domain (78). Until now, researchers have not identified a certain receptor for B7-H4. While researchers considered a B and T lymphocyte attenuator as a B7-H4 receptor, this has not been supported by additional experiments (78). B7-H4 mRNA presents a wide distribution in normal tissues, however, it has a limited expression in cancer (79). In normal tissue, B7-H4 mRNA is expressed on bone marrow-derived DCs, APCs, B cells, peritoneal macrophages and widely distributed in non-lymphoid tissue. Different with other members, B7-H4 exhibits a strict expression on cells originate in hematopoietic. *In vitro* culture, B7-H4 expression is lost rapidly (80). B7-H4 protein has been found to overexpress in several cancer tissue, including ovarian, pancreatic cancer, renal cell cancer, hepatocellular carcinoma (HCC), gastric cancer, glioma, lung cancer, breast, prostate cancer, cervical cancer and melanoma (81). Studies indicate that cytokines can effectively regulate B7-H4. B7-H4 expression can be increased by IL-10 and IL-6, but decreased by IL-4 and DC-differentiation cytokines (82, 83). Yao et al. found that IL10 and IL6 produced by CD133+ cells induce B7-H4 expression by glioma-infiltrating macrophages (84). According to Zhou et al., B7-H4 expression in mouse tumor cells decreases IFN-γ production and negatively regulates the cytotoxicity, expansion, and activation of CD8 tumor-specific T cells. This process can promote tumor growth and weaken tumor-specific immunity (85). Studies suggest that recombinant anti-B7-H4 antibodies may assist in enhancing anti-tumor immune responses as well as triggering T-cell activation (80, 86). In human glioma, B7-H4 expression shows a positive association with advanced glioma grade and poor prognosis (87). Yao et al. detected B7-H4 mRNA and protein expression in glioma tissue and showed that levels increased as the disease progressed. B7-H4 can be a prognostic marker for glioma. IL-6 increases B7-H4 expression by activating the IL-6/JAK/STAT signaling pathway (84). **Figure 4** displays the regulatory actions of B7-H4 expression. In a xenograft glioma model, T cells become activated if the B7-H4 gene is silenced, hence it could serve as a possible target for glioma therapy. To determine coexpression levels of PD-L1 and B7-H4, two primary B7 immune regulatory molecules, in glioma, Chen et al. adopted immunohistochemistry (IHC) for assessing 505 tumor tissues of primary gliomas (stage II–IV) and found that 23% and 20% of patients expressed PD-L1 and B7-H4, respectively, while only 2% of patients co-expressed the two proteins (88). These findings demonstrate that PD-L1 and B7-H4 may be mutually compensatory immune checkpoint molecules for immune targeted or activation-specific immunotherapy against gliomas. In addition, based on an exploratory randomized phase II clinic trial, GBM patients who had low B7-H4 expression had obviously longer OS after receiving a dendritic cell vaccine (DCV) (89). B7-H4 could help to predict the success of this treatment in glioma patients.

B7-H4 expressed in various human cancer, and that its overexpression serves as adverse prognostic marker that significantly correlated with patient’s poor prognosis makes it an attractive drug target. B7-H4 can be targeted through a variety of mechanisms like monoclonal-blocking antibodies (mAbs), antibody–drug conjugate (ADCs), CD3 bispecific antibodies (BiTE), single chain fragment variables (scFvs) and CAR-T (80, 90, 91). Since ovarian cancer sees the expression of B7-H4, anti-B7-H4 scFvs have shown the ability to delay the growth of established ovarian cancer (80). A B7x *scFv/CD3 BiTE* has been shown it strong antitumor activity in preclinical breast cancer model to control the growth of breast cancer cell line (92). In addition, B7-H4 specific target CAR-T cells ability of recognizing both murine and human B7-H4 led to tumor regression in xenograft models (90). Regretfully, to date, there is no ongoing or finished clinical/preclinical trial targeting B7-H4 in glioma.

## 6 Clinical meanings and functions of B7-H6 in gliomas

**Figure 5:**
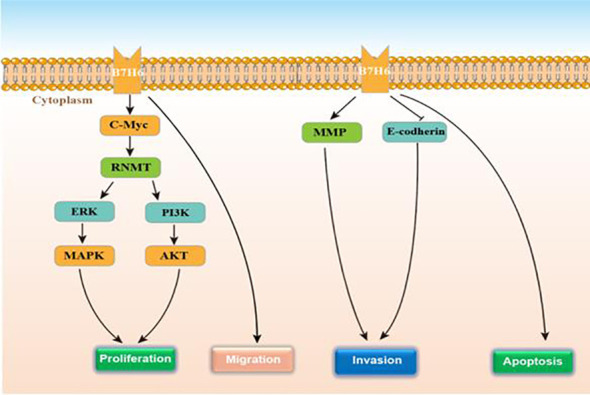
The function and regulatory mechanisms of B7-H6 in gliomas.

B7 homolog 6 (B7-H6), also called NCR3LG1, is a kind of the immune checkpoints of the B7 family and plays the role of an endogenous/co-stimulatory ligand. This gene encodes a 454-aa-long type I transmembrane protein of which the predicted molecular mass is 51 kDa (93). The B7-H6 extracellular region contains both an IgV-like and an IgC-like domain. Using a residue mutation strategy, Gordon Joyce et al. found that there is a direct and selective interaction between the extracellular domain of NKp30, an NK cell-activating receptor, and the B7-H6 extracellular domain (94). Upon binding to its receptor, NKp30 becomes immunogenic and induces NK cell immunosurveillance. The intracytoplasmic domain has many signaling motifs, e.g. an inhibition motif based on immunoreceptor tyrosine (SaYtpL), a SH2 (Src homology 2)-binding domain (YqlQ), and a SH3-binding motif (PdaPilPvsP) (95). B7-H6 also presents a selective expression on several tumor cell types (melanoma, neuroblastoma, primary blood or bone marrow cells from various hematological malignancies, etc. (96, 97). This protein is undetectable in normal tissue and normal peripheral blood mononuclear cells. Researchers have explored B7-H6 expression and regulation mechanism. One study investigated the induction of B7-H6 at the surface of neutrophils and proinflammatory monocytes with ligands of TLR and proinflammatory cytokines (TNF-α and IL-1β) (98). In another study, B7-H6 expression on the tumor cells surface was triggered by metalloproteases and the regulation relied on siRNA-mediated gene attenuation or metalloprotease inhibitors, which increased B7-H6 expression and strengthened NKp30-mediated NK cell activation (99). According to the study by Guo et al. in 2016, B7-H6 presents an over-expression in human astrocytoma tissues, and is positively correlated with WHO tumor grade (100). Jiang et al. found that B7-H6 remarkably regulated the biological behavior of glioma cells. Knocking the B7-H6 down in glioma cells, the cell proliferation, migration, and invasion were obviously suppressed, however, the apoptosis and cell cycle arrest were strengthened (101). Conforming to these findings, Che et al. revealed that B7-H6 knockdown in the glioma cell exerted an obvious increased effect on the expression of X protein associated with E-cadherin and Bcl-2, as well as suppressed the expressions of vimentin, matrix metalloproteinase-2, N-cadherin, matrix metalloproteinase-9 and survivin expression (102). They also found that lipopolysaccharide (LPS) could induce B7-H6 expression in glioma cells. To better understand how B7-H6 expression affected the tumor tissue of glioma from biological perspective, Chen et al. conducted a study. They found the high expression of B7-H6 in GSLCs from the glioma cell lines *in vitro*. Interestingly, among the B7 family members, B7-H6 was the only member with preferential expression in the GSLCs. They also found that GSLC proliferation was promoted by PI3K/Akt and ERK/MAPK and c-Myc/RNMT axis signaling pathways (38). Wu et al. found that B7-H6 knockdown remarkably restricted the tumorigenesis as well as facilitated the chemosensitivity through STAT3 signaling pathway in B-cell non-Hodgkin lymphoma, which may provide us with some enlightenments on investigating on chemosensitivity of glioma (103). **Figure 5** displays the regulatory actions of B7-H6 expression. Therefore, B7-H6 can effectively mark glioma diagnosis and prognosis from biological level, and is a useful target for new treatment therapy.

B7-H6 presents an obvious expression in various cancer types, it therefore serves as a proper candidate for targeted treatment. Researchers consider the utilization of certain monoclonal antibodies against B7-H6 as an effective method for tumor treatment. Gacerez et al. has revealed that mouse scFv-based CARs can target B7-H6 in Lymphoma, thereby enhancing the T cells’ anti-tumor activity( (104). Regretfully, so far, there is no ongoing or finished clinical/preclinical trial targeting B7-H6 in glioma.

## 7 Conclusion

In the last decade, a lot of ICIs targeting B7 family have been developed and tested in various solid cancers. However, the role of B7 family members in glioma remains largely unexplored. Thus, further understating of the mechanism and function of the B7 family in glioma would contribute to discovering more effective immunotherapy targets. The current study demonstrated that B7-H3 and B7-H5 presented higher expression than other family members, suggesting these two molecules may play an essential role in anti-glioma immunity. Additionally, the nature of glioma as a “cold” tumor severely restrict the effect of ICIs. Future research should also focus on how to reverse the immunosuppressive microenvironment in glioma.

## Author contributions

YW took charge of study design and manuscript writing. ML, GW and HW were responsible for manuscript editing and revision. The submitted version has obtained the approval of all authors.
